# Risk Prediction Model for Uncontrolled Hypertension in Chinese Community

**DOI:** 10.3389/fcvm.2021.808071

**Published:** 2022-01-24

**Authors:** Zhiping Gao, Shiqun Chen, Xiaoyu Huang, Jianfeng Ye, Jin Liu, Zhidong Huang, Jiyan Chen, Liwen Li, Yong Liu, Shuguang Lin

**Affiliations:** ^1^Guangdong Cardiovascular Institute, Guangdong Provincial People's Hospital, Guangdong Academy of Medical Sciences, Guangzhou, China; ^2^Department of Cardiology, Guangdong Cardiovascular Institute, Guangdong Provincial People's Hospital, Guangdong Academy of Medical Sciences, Guangzhou, China; ^3^Department of Guangdong Provincial Key Laboratory of Coronary Heart Disease Prevention, Guangdong Cardiovascular Institute, Guangdong Provincial People's Hospital, Guangdong Academy of Medical Sciences, Guangzhou, China; ^4^Department of Cardiology, Yangjiang People's Hospital, Yangjiang, China; ^5^Department of Cardiology, Dongguan TCM Hospital, Dongguan, China

**Keywords:** uncontrolled hypertension, screen, risk factor, prediction model, community

## Abstract

**Background:**

Uncontrolled hypertension rate was still high across China. This study develops and validates an index to help quantify the combination of socio-behavioral aspects to screen high-risk patients in uncontrolled hypertension in Chinese primary care.

**Methods:**

A cross-sectional study included 1,039 of patients with hypertension in the Chinese community. We assessed independent risk factors of uncontrolled blood pressure (defined as having a blood pressure ≥140/90 mmHg, even with antihypertensive therapy) and develop a risk prediction model.

**Results:**

Among the 1,039 patients (53.9% male, the average age was 61 ± 13 years), 452 (43.5%) were uncontrolled hypertensive. Multivariable analysis showed that worker (odds ratio, OR: 1.98, 95% CI: 1.46–2.69), no health insurance (OR: 3.47, 95% CI: 2.08–5.80), non-marital status (OR: 2.01, 95% CI: 1.35–3.27), and other socio-behavioral aspects were independent risk factors of uncontrolled hypertension, which were included the final prediction model (C-static: 0.781). With internal validation by the bootstrap method, the risk score showed good discriminating ability and predicting ability for the incidence of uncontrolled hypertension (C-static: 0.771).

**Conclusions:**

This study showed that nearly half of the patients suffered from uncontrolled hypertension in the Chinese community. We established a prediction model with good predictability to help quantify the combination of socio-behavioral aspects and screen high-risk patients with uncontrolled hypertension.

## Introduction

Hypertension is an important health challenge worldwide because of its high prevalence, leading to cardiovascular disease, premature death, and disability (1, 2). National reports have indicated that the unawareness and uncontrol of hypertension improved substantially in high-income countries, while there has been little improvement in low- and middle-income countries (3). In China, the prevalence of hypertension in adults is gradually increasing, reaching 27.9%, but the control rate is only 15.3% (4).

Previous studies have shown that China cardiovascular outpatient clinics of comprehensive second- and third-level hospitals show that the protective factors of blood pressure control rate include older, retirees, medical care, physical activity, and isolated hypertension patients (5). Patients in economically developed areas have a high accuracy rate of self-reported hypertension. The American Health Nutrition Survey shows that elderly, obesity, and diabetes are independent risk factors for controlled hypertension. The characteristics and rates of controlled hypertension in general hospitals and communities are different (6).

The risk assessment of uncontrolled hypertension in community hypertension patients can help identify high-risk patients and carry out active blood pressure control measures for target factors. The recent systematic review showed a moderate to large effect of patients' education (healthy knowledge and behavior) on adherence to lifestyle modifications and blood pressure control (7). However, there was a lack of an index to help quantify the combination of socio-behavioral aspects, such as knowledge of hypertension and medicine adherence, which can screen high-risk patients in uncontrolled hypertension in Chinese community clinics.

Therefore, we aimed to develop and validate a useful risk prediction for uncontrolled hypertension among patients receiving antihypertensive therapy in Chinese community primary care.

## Methods

### Data Sources and Study Population

**Figure 1:**
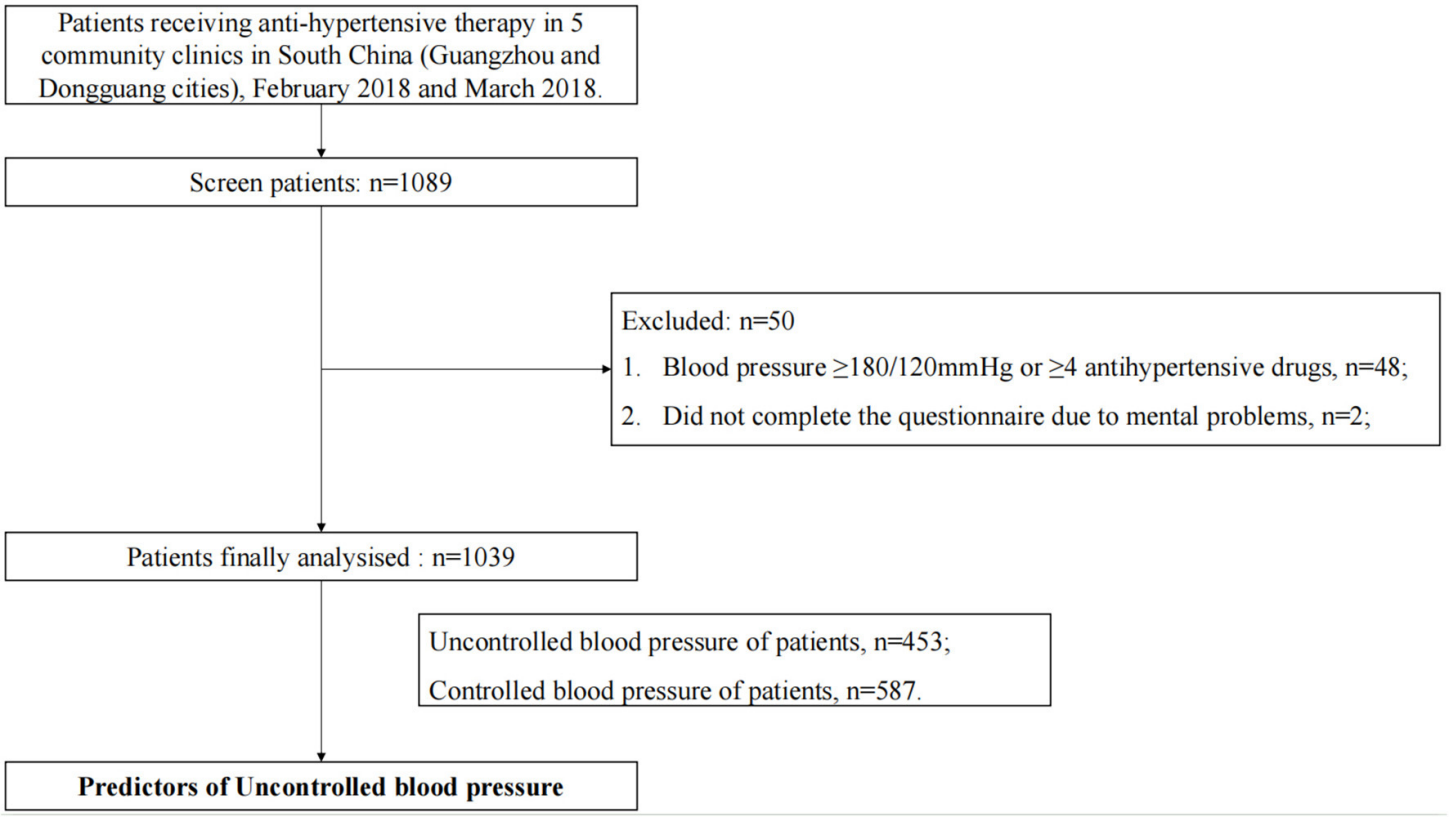
Study flow.

A total of 1,089 patients with a history of hypertension and receiving antihypertensive medicines who went to the community hypertension clinic were consecutively enrolled from 5 communities within a limited time window (1 week) in Guangzhou and Dongguan, China, between February 2018 and March 2018, participated in this survey. We exclude patients <18 years old; patients with blood pressure ≥180/120 mmHg or antihypertensive medicines ≥4 (definitely recommend they seek treatment from a specialist) (*n* = 48); Patients unable to complete the questionnaire due to mental problems (*n* = 2). Finally, 1,039 patients were included in the final analysis (Figure 1). Our study obtained written informed consent from all patients in compliance with the Declaration of Helsinki (approved by the Ethics Research Committee of Guangdong Provincial People's Hospital) (8).

### Data Collection

We collected the social-demographic information, disease awareness, hypertension management, and the use and demand of mobile health tools in patients with hypertension in the community primary care in person, which was previously described (9). All patients completed the survey, and the average time it took participants to complete the survey was 20 min. Sex, age, height, weight, education (International Standard Classification of Education, ISCED), occupation, and medical care were evaluated by standard survey items (Supplementary Materials).

### Definitions

Uncontrolled blood pressure is defined as having a blood pressure ≥140/90 mmHg, even with antihypertensive therapy. The blood pressure was measured after the patient sat still for a while, with at least two measurements, with an interval of 1–2 min (if the previous two measurements are very different, additional measurements will be taken). The patient's marital status of either unmarried, divorced, or widowed is defined as non-marital status. Working full-time was defined as the total daily working hours of the patient of more than 8 h. Patients with a clear definition of hypertension and understanding of the risks of hypertension were defined as having the correct knowledge of hypertension treatment. Patients who take medication regularly, monitor blood pressure regularly, and spend more than 0.5 h of daily activity in total are defined as right knowledge of hypertension treatment. Good medication compliance was defined as missed medication less than once a week. The willingness to remind blood pressure measurement information is defined as having been using or willing to accept health software or SMS reminders as an auxiliary tool to regularly measure blood pressure. Further detailed definitions are provided in Supplementary Materials.

### Statistical Analyses

We estimated that 10 risk factors would be included in the multivariate regression model. In regression analysis, the sample size used for prognostic risk factor analysis requires at least 20 individuals uncontrolled hypertension (events) for each prognostic factor, so we need 200 total events. The rate of uncontrolled hypertension in patients with hypertension is not <20%. Accordingly, a total of 100 samples are needed.

We compared patients with and without uncontrolled blood pressure. Continuous variables were reported as the mean and SD, categorical variables were described as frequencies and percentages and compared using the χ^2^ test. Clinical potential confounders, the baseline variables with differences, and value of *p* < 0.05 in the univariate logistic analyses were regarded as candidate covariates. The variables with *p* < 0.05 in logistic regression multivariate analysis, including candidate covariates, are independent risk factors that affect uncontrolled blood pressure. The risk factors that are important in univariate analysis can be used to select the final prediction model for uncontrolled hypertension. Full-time work, no medical care insurance, non-marital status, poor cognition of hypertension diagnosis, poor drug compliance, poor cognition of hypertension treatment, and unwillingness to accept informational intervention were identified as independent predictors of blood pressure in the community with hypertension. The modeling data set of 906 community hypertension patients was used, and the final prediction model was assessed using the area under the receiver operating characteristic (ROC) curve and concordance c-statistic for discriminative ability, and the Hosmer–Lemeshow goodness-of-fit statistic for calibration using fifths of the fitted risk values (10). Moreover, the final model was tested by the bootstrapping method (1,000 times) to evaluate the stability of the c-statistics. The statistical analysis was conducted using SAS V.9.4.

## Results

Totally 1,039 patients were included in the final analysis. The average age was 61 ± 13 years old, there were 549 (53.9%) male, the average body mass index was 24.3 ± 3.2 kg/*m*^2^, and 153 (15%) were unmarried, the total uncontrolled hypertension rate (≥140/90 mm Hg) 452 (43.5%). Compared with patients with controlled hypertension, patients with uncontrolled hypertension and receiving antihypertensive treatment were younger (59 vs. 62 years), more likely to be unmarried (19.5% vs. 12.3%), full-time employed (51.1 vs. 37.5%), and have self-financed medical care (17.6 vs. 5.0%). However, they have less knowledge of hypertension diagnosis (41.2 vs. 72.7%), hypertension treatment (31.4 vs. 60%), and hypertension medications (56.9 vs. 77.5%). In addition, they were less complied with antihypertensive medications (22.9 vs. 38%), and less willing to remind of blood pressure measurements (56.9 vs. 77.5%), more smoke (40.1 vs. 32.9%), and less exercise (69.3 vs. 55.9%) (*p* < 0.05 above), but no difference in gender, body mass index, blood pressure measurement compliance, drug purchase location, etc., (Table 1).

**Table 1:** Sample characteristics of uncontrolled hypertension patients.

**Item**	**Missing**	**Total sample**	**Controlled hypertension**	**Uncontrolled hypertension**	***P***
		***N* = 1,039**	***N* = 587 (56.5)**	***N* = 452 (43.5)**	
**Age (SD)**	22	61 (13)	62 (13)	59 (12)	<0.001
**Age > 75 years**, ***n*** **(%)**	22	161 (15.83)	106 (18.47)	55 (12.42)	0.009
**Male**, ***n*** **(%)**	21	549 (53.9)	303 (53.06)	246 (55.03)	0.53
**BMI (SD)**	51	24.3 (3.2)	24.4 (3.1)	24.1 (3.4)	0.092
**Non-marital status**, ***n*** **(%)**	7	153 (14.7)	72 (12.3)	88 (19.5)	<0.001
**Employment status, not working**, ***n*** **(%)**	7	451 (43.41)	220 (37.5)	231 (51.1)	<0.001
**No medical insurance**, ***n*** **(%)**	15	108 (10.5)	29 (5.0)	79 (17.6)	<0.001
**Good knowledge of hypertension diagnosis**, ***n*** **(%)**	5	613 (59)	427 (72.7)	186 (41.2)	<0.001
**Good knowledge of hypertension treatment**, ***n*** **(%)**	16	494 (47.6)	352 (60.0)	142 (31.4)	<0.001
**Good knowledge of anti-hypertension medicine**, ***n*** **(%)**	21	712 (68.5)	455 (77.5)	257 (56.9)	<0.001
**Good BP monitoring**, ***n*** **(%)**	19	387 (37.25)	223 (38.0)	164 (36.3)	0.57
**Good medicine adherence**, ***n*** **(%)**	4	325 (31.4)	222 (38.0)	103 (22.9)	<0.001
**Willing to remind medication information**, ***n*** **(%)**	225	198 (19.1)	121 (20.6)	77 (17.0)	0.145
**Willing to remind blood pressure measurement information**, ***n*** **(%)**	225	162 (15.6)	106 (18.1)	56 (12.4)	0.012
**Smoking**, ***n*** **(%)**	38	395 (39.5)	186 (32.9)	209 (40.1)	<0.001
**Weekly high-intensity exercise**, ***n*** **(%)**	226	641 (61.7)	328 (55.9)	313 (69.3)	<0.001
**Wechat used by patients**, ***n*** **(%)**	217	128 (12.3)	66 (11.3)	62 (13.7)	0.22
**Wechat used by patients' family**, ***n*** **(%)**	222	455 (43.8)	242 (41.2)	213 (47.1)	0.06
**Medicine prescribed in above second-class hospital**, ***n*** **(%)**	4	230 (22.1)	119 (20.3)	111 (24.6)	0.1

**Figure 2:**
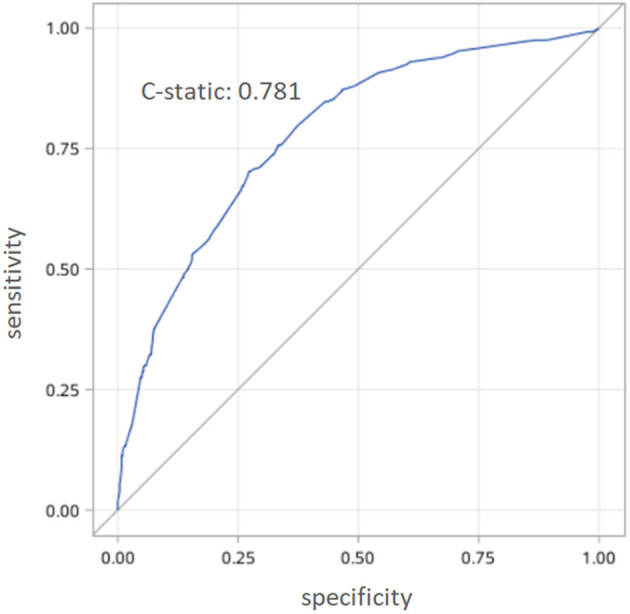
The receiver operating characteristic (ROC) analyses of the prediction model.

Univariate analysis results were shown in Table 2. Multivariable analysis showed that: full-time work [odds ratio (OR): 1.98, 95% CI: 1.46–2.69], self-financed medical care (OR: 3.47, 95% CI: 2.08–5.80), non-marital status (OR: 2.01, 95% CI: 1.35–3.27), poor knowledge of hypertension diagnosis (OR: 3.28, 95% CI: 2.42–4.45), poor drug compliance (OR: 1.51, 95% CI: 1.08–2.11), poor knowledge of hypertension treatment (OR: 2.94, 95% CI: 2.16–3.99), and reluctance to remind blood pressure measurement information (OR: 1.64, 95% CI: 1.08–2.50) were identified independent predictors of uncontrolled hypertension among patients in the community who received antihypertensive treatment (Table 3). The receiver operating characteristic (ROC) curve analysis showed the area under the ROC curve was 0.781 (Figure 2).

**Table 2:** Logistics univariate analysis of substandard blood pressure control.

**Variables**	**vs**.	**OR**	**CI**	***P* value**
**Age > 75 years**	≥75 vs. <75	0.63	0.44–0.89	<0.01
**Marital status**	Non-marital status status vs. marital status status	1.92	1.35–2.71	0.0002
**Employment status**	Full time working vs. not working	1.74	1.36–2.24	<0.001
**Medical insurance**	Self-financed vs. others	4.04	2.59–6.31	<0.001
**Knowledge of hypertension diagnosis**	poor vs. well	3.82	2.94–4.95	<0.001
**Knowledge of anti-hypertension medicine**	poor vs. well	2.62	2.00–3.42	<0.001
**BP monitoring**	Good vs. not	0.93	0.72–1.20	0.573
**Willing to remind medication information**	Yes vs. not	0.791	0.58–1.09	0.15
**Willing to remind blood pressure measurement information**	Yes vs. not	0.64	0.45–0.91	0.01
**Weekly high-intensity exercise**	Enough vs. no	1.78	1.37–2.30	<0.001
**Smoking**	Yes vs. no	1.89	1.46–2.44	<0.001
**Wechat used by patients**	Yes vs. not	1.26	0.87–1.82	0.23
**Wechat used by patients' family**	Yes vs. not	1.27	0.99–1.63	0.06
**Medicine adherence**	Good vs. not	0.49	0.37–0.64	<0.001
**Medicine prescribed in above second-class hospital**	second-class hospital and above vs. others	1.28	0.95–1.72	0.1
**Knowledge of hypertension treatment**	Well vs. poor	0.31	0.24–0.40	<0.001
**Daily measurement of BP**	Yes vs. not	0.59	0.42–0.83	0.003
**BMI**	/	0.97	0.93–1.01	0.09

**Table 3:** Multivariate analysis of risk factors of drug non-compliance in patients with hypertension in community.

**Risk factors**	**Test group vs. reference group**	**OR**	**95%CI**	***P***	**Weighted integral**
Employment status	Full time working vs. others	1.98	1.46–2.69	<0.001	4
Medical insurance	Self-financed vs. medical insurance	3.47	2.08–5.80	<0.001	7
Marital status	Non-marital status vs. married	2.01	1.35–3.27	<0.001	4
Knowledge of hypertension diagnosis	Poor vs. well	3.28	2.42–4.45	<0.001	6
Medicine adherence	Bad vs. good	1.51	1.08–2.11	0.016	3
Knowledge of hypertension treatment	Wrong vs. right	2.94	2.16–3.99	<0.001	6
Willing to remind blood pressure measurement information	Not vs. yes	1.64	1.08–2.50	0.02	3

**Figure 3:**
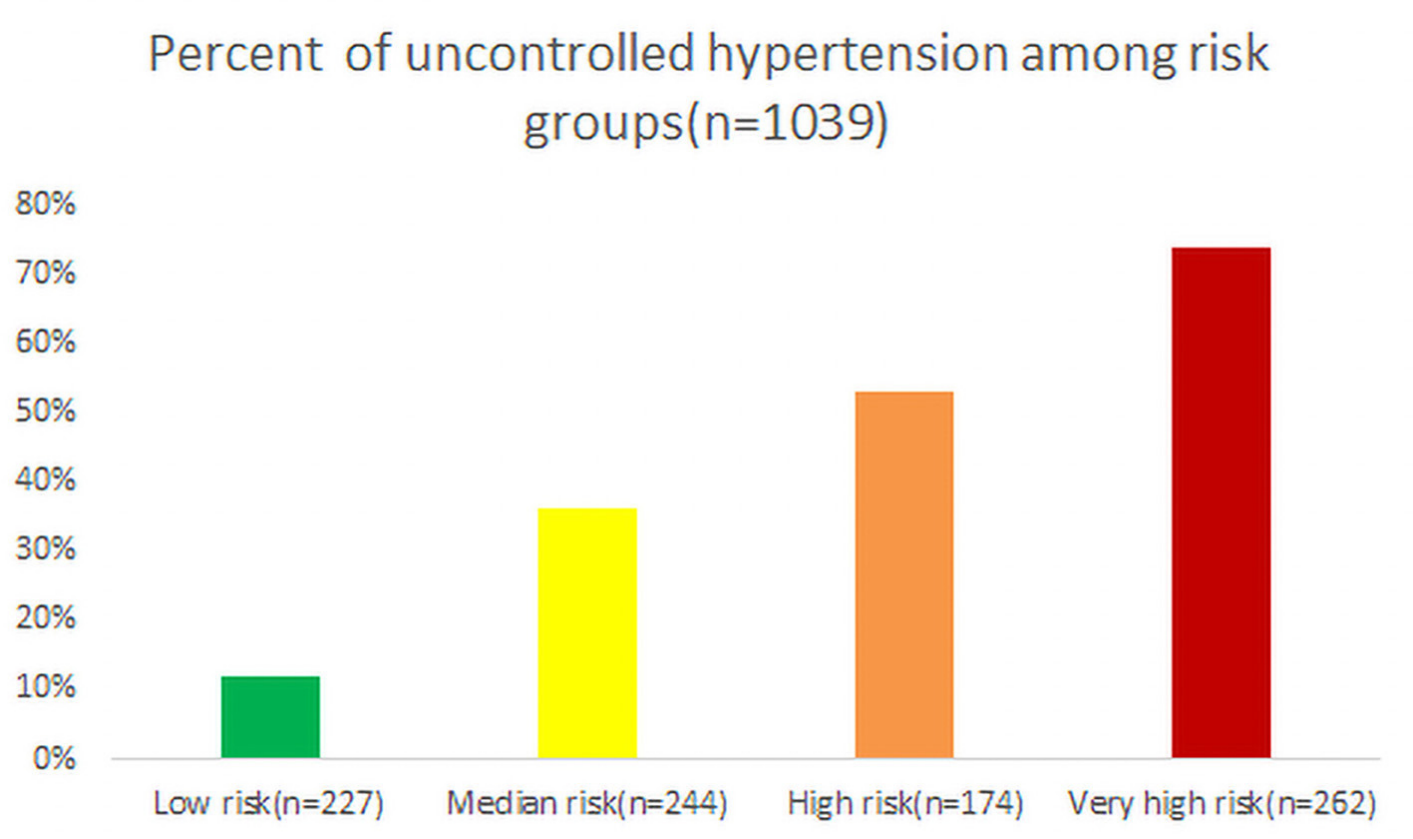
Uncontrolled hypertension with different risk scores.

The risk model includes the following seven factors: full-time work (4 points), self-pay medical care (7 points), non-marital status (4 points), poor cognitive diagnosis of hypertension (6 points), poor drug compliance (3 points), poor cognition of hypertension treatment (6 points), and unwilling reminder of blood pressure measurement (3 points) (Table 3), Hosmer Lemeshow statistics of multivariate models do not suggest lack of appropriateness (*x*^2^ = 6.5649, *P* = 0.5842). Based on the frequency of uncontrolled hypertension with different risk scores, 1,039 patients were classified into three groups in the modeling data set: low risk [ <7 points, *n* = 227 (12%)], moderate risk [8–14 points, *n* = 244 (36%)], high risk [15–22 points, *n* = 174 (53%)], and extremely high risk [>22 points, *n* = 262 (74%)] (Figure 3). In the verification data set, the risk score showed good discriminating ability and predicting ability for the incidence of uncontrolled hypertension (C-static: 0.771) by the bootstrap method.

## Discussion

To the best of our knowledge, this study was the first study to estimate a risk-prediction model for uncontrolled hypertension in the Chinese community. Our data suggested that nearly half of the hypertension patients who have received antihypertensive treatment in the community suffered from uncontrolled hypertension. Bad hypertension health cognition and lifestyle behavior were independent risk factors for uncontrolled hypertension. We have established a prediction model based on 7 key predictors of uncontrolled hypertension among community patients receiving antihypertensive treatment, with good predictability and high discriminatory ability.

This study found the uncontrolled hypertension rate was 43.5% (in 2008), in the developed region in South China, in line with recent trends of the United States, where near half of patients achieved hypertension control across 20 American primary care centers, and the hypertension control increased from 27.3% in 1988–1994 to 50.1% in 2007–2008 in recent NHANES survey (10–12). Wang et al. conducted MMM China project among 364,000 participants in 394 inside hospitals or community health centers and found that the awareness, treatment, and control rates of hypertension were 60.1, 42.5, and 25.4%, respectively, with the rate of uncontrolled hypertension (59.8%) moderately higher than our study (13). We only investigated the patients receiving antihypertensive treatment in the community, not patients in hospitals, which might present with higher control proportion.

The patients with uncontrolled hypertension in our study were younger, more unmarried, have more full-time work, more self-paying medical treatment, less knowledge of hypertension, less medication compliance, more smoking, and less exercise.

Our analysis also showed that the independent risk factors of uncontrolled hypertension are full-time work, self-financed medical care, non-marital status, poor cognition of hypertension diagnosis, poor cognition of hypertension treatment, poor drug compliance, and unwillingness for blood pressure measurement information reminder among patients in the community receiving antihypertensive treatment. MMM China project also showed that current smoking, no diabetes, no coronary heart disease, and older were independent risk factors of uncontrolled hypertension (13), while the above variables were not collected in our study. A recent review highlighted the important role of an education intervention on healthy behaviors and showed a moderate effect in the adherence to dietary recommendations and physical activity recommendations (7). Finnish Public Sector study with 41,225 participants suggested that those hypertensive patients were less likely to adhere to lifestyle modifications after the initiation of medications, as evidence of an increasingly becoming obese and physically inactive (14).

The WHO recommended more attention on nursing in the community, which could greatly contribute to many kinds of population groups in the community. Because of the role of a healthy lifestyle in hypertension control, we need to explore effective components of lifestyle modification educational intervention in terms of better delivery mode, use of the theoretical framework, and use of supportive methods. Theory-based educational interventions, such as knowledge of hypertension diagnosis, hypertension treatment, and consistent measures of adherence behavior, such as medicine compliance, blood pressure measurement, are needed to be adopted in future practice or studies. Monthly or weekly group education can be conducted in the community to promote hypertension control, especially for the patients on working or patients under non-marital status.

Besides the patients under treatment, we also should emphasize the inadequate use of antihypertensive treatment in the community in China. A combination of antihypertensive therapy would be a choice of approaches for improving control of hypertension in China. (13). Wang et al. established a web-based and WeChat-linked blood pressure measuring system in China mainly in public areas, such as office buildings, shopping malls, airports, railway stations, and so on, where younger people often walk around (15). Adherence to blood pressure measurement was a barrier to hypertension control. However, there were knowledge gaps, such as appropriate arm and body positioning, frequency of readings, the timing of measurements, duration of rest before measurement, proper cuff size and placement, the necessity of voiding before measurement, and the importance of refraining from other activities when obtaining reading (16). Canada study showed that only 8% of patients with hypertension were trained with the home blood pressure monitoring (HBPM) technique (17). Another American study with the HBPM program found that 13% of patients were sufficiently compliant with BP measurement guidelines to ensure reliable readings (18). Similarly, one Chinese cross-sectional survey collected data among 2,272 patients with hypertension aged ≥35 years from 20 communities across three cities and six townships in three provinces and found that only 45.3% owned a home blood pressure (BP) monitor. In addition, ~4.4% of participants had achieved optimal HBPM method (duplicate measurements in the morning and evening for 1 week), and only 16.0% of participants actively reported their HBPM readings to doctors (19).

We have established a prediction model based on 7 key predictors of uncontrolled hypertension among community patients receiving antihypertensive treatment, with good predictability and high discriminatory ability. A recent review suggested that mHealth apps can be beneficial in terms of improving hypertension self-assessment, treatment, and control (20). One American study used electronic medical record (EMR) data from patients at two urban safety-net clinical systems and suggested that stable insurance of any type was associated with better hypertension control than no or unstable insurance. Therefore, we should pay more attention to hypertensive patients without health insurance and provide more economic antihypertension medicines. Our prediction model provides a good evaluation tool for community hypertension prevention and treatment to identify high-risk populations of uncontrolled hypertension, but still, needs evaluation and external validation/clinical promotion in future large-scale multicenter trials. We provided a new type of intervention target (hypertension health cognition and lifestyle behavior) for community hypertension prevention and control. Future studies, including large-scale randomized clinical trials with patients-centered design, are crucial to further evaluate the potential and effectiveness of interventions, such as mHealth apps-based patients' education, community levels, or integrate interventions in the hypertension control in the community.

## Limitation

First, this study was a cross-sectional survey in the developed region (Guangzhou city and Dongguan city), which lacked the outcome among these participants, such as cardiovascular events and could not investigate associations of real office-BP control and their characteristics, while survey with questionnaire characteristics may be easier to recruit more patients in Chinese community economically. Second, there is a lack of information about important comorbidities that may affect blood pressure, such as drug use, kidney failure, or diabetes. This information may give us a better understanding of blood pressure. Third, the subjects were patients under anti-hypertension treatment, missing the information among patients without treatment in primary care practice or patients prescribed outside the primary care. But we recruited most the hypertensive participants in the clinic of primary care or community resident with hypertension by the following phones. Fourth, the risk factors in this study were defined in the questionnaire, such as evaluating the knowledge of the patient on hypertension, diagnostic criteria, hypertension drug, hypertension complication, etc. according to the subjective assessment of the patient, but the study found that the hypertension health knowledge level of hypertension patients is closely related to hypertension standards, defining new risk factors as future community hypertension control to provide intervention targets. Fifth, because we only recruited patients in 5 urban communities and communities are not randomly sampled from the general community, our results cannot be generalized to all communities (e.g., rural areas).

## Conclusion

This study showed that nearly half of the patients receiving antihypertensive treatment suffered from uncontrolled hypertension in the Chinese developed community. We also found the independent risk factors for uncontrolled hypertension, including full-time work, self-financed medical care, non-marital status, poor cognition of hypertension diagnosis, poor cognition of hypertension treatment, poor drug compliance, and unwillingness for blood pressure measurement information reminder, and established a prediction model based on 7 keys predictors of uncontrolled hypertension, with good predictability and high discriminatory ability. Our prediction model provides a good evaluation tool for community hypertension prevention and treatment to identify high-risk populations of uncontrolled hypertension, but still, needs evaluation and external validation/clinical promotion in future large-scale studies.

## Data Availability Statement

The original contributions presented in the study are included in the article/Supplementary Material, further inquiries can be directed to the corresponding author/s.

## Ethics Statement

All the traceable personal identifiers were removed from the analytic dataset to protect patients' privacy. The study protocol was approved by the Guangdong Provincial People's Hospital Ethics Committee, and this study was performed according to the Declaration of Helsinki.

## Author Contributions

ZG, SC, YL, and SL contributed to conception and design. ZG, SC, and YL contributed to manuscript writing. ZG, SC, YL, JY, JL, and ZH contributed to administrative support. All authors contributed to provision of study materials or patients, collection and assembly of data, data analysis and interpretation, and approval of final version of the manuscript.

## Conflict of Interest

The authors declare that the research was conducted in the absence of any commercial or financial relationships that could be construed as a potential conflict of interest.

## Publisher's Note

All claims expressed in this article are solely those of the authors and do not necessarily represent those of their affiliated organizations, or those of the publisher, the editors and the reviewers. Any product that may be evaluated in this article, or claim that may be made by its manufacturer, is not guaranteed or endorsed by the publisher.
