# Supplementary material for: Risk Prediction Model for Uncontrolled Hypertension in Chinese Community
Source: Front Cardiovasc Med. 2022 Jan 24;8:808071. doi: 10.3389/fcvm.2021.808071 (PMC8818737; doi:10.3389/fcvm.2021.808071)
Supplement: Supplementary file 1 [file Table_1.DOCX]

**English language version**

Questionnaire for community hypertension patients

Part 1: Personal information

1. Your gender is:

A. male B. Female

1. Your date of birth is
2. Your height is cm, and your weight is cm.
3. Your marital status is:

A. married B. unmarried C. divorced D. widowed

1. Your cultural education level is:

A. elementary school and below B. junior high school

C. high school D. university and above

1. What are your working hours every day?

A. not working B. less than 4 hours C. 4-8 hours D. More than 8 hours

1. 7. How will your medical expenses be paid?

A. Residents' medical insurance B. Social Insurance C. New rural cooperative medical care D. Employee insurance or free medical care

E. personal expenses f. children's payment G. Others

Part 2: Hypertension management conditions

1. What do you think is the value of blood pressure to diagnose hypertension in Chinese adults? A. Unclear

B. Systolic blood pressure ≥ 150mmHg and or diastolic blood pressure ≥ 90 mmHg

C. Systolic blood pressure ≥ 140mmHg and or diastolic blood pressure ≥ 90 mmHg

1. Do you think that hypertension can cause myocardial infarction, stroke or renal impairment ? A. Yes B. No C. Don't know
2. What is your blood pressure situation?

A. measure at home, but not self-adjusting.

B. measure at home and adjusting the drug according to blood pressure value

C. measure only in the hospital office

1. Please answer the following questions about medication with “Yes” or “no”
2. Did you take the medicine yesterday?
3. Have you forgotten to take the medicine in the past two weeks?
4. Did you forget to take the medicine?
5. When you feel that your symptoms are worse or other discomfort, do you increase your dose or change your medication?
6. Have you ever forgotten to take your medicine with you when you are traveling or leaving home for a long time?
7. When you feel that your blood pressure has been controlled, have you stopped taking the medicine?
8. Is it difficult for you to take medication and measure blood pressure as required?
9. Did you forget the time and amount of medication?
10. Your smoking status is:

A. Never smoke B. Still smoking C. Smoking in the past and quit now

1. You drinking habit is:

A. Drinking a large amount (an average of 1 white liquor per day, or a relatively alcoholic wine or beer)

B. Drinking a small amount (less than A daily alcohol consumption)

C. Never drinking

1. How do you exercise every day?

A. Inactivity B. less than 0.5 hours C.0.5-1 hours D.1-2 hours E. More than 2 hours

1. Is your diet balanced?

A. Balance (each with vegetables, fruits and grains)

B. Unbalanced

1. You think that your salt intake is:

A. Salty: Daily salt intake per person ≥ 6 grams (one beer bottle cap *), pay attention to the intake of hidden salt (salty, chicken, soy sauce, etc.)

B. Less salt (not meeting the above criteria)

10、Do you think you are a low-fat diet (rarely eat animal fat, fried foods, junk food, etc.)?

A. Yes B. No.

Part 3: The use of WeChat and mobile health apps

1. Have you used WeChat in the past year? Yes or No
2. Have you used health applications (apps, applets) in the past year? Yes or No
3. What health behaviors do you want to promote by health applications?

A. Quitting smoking B. exercise C weight loss D. balanced diet

E. alcohol withdrawal F. others

1. What features does the health application you use have?

A. provision of information on the target behavior

B. motivational messages

C. goal setting and action planning

D. Reminder

E. information on the current status and individual progress

F. I do not use any of these app characteristics

1. Are you willing to use the hypertension management application?

A. Already using B. Willing C. Unwilling

1. What is your reason for using the hypertension management application?

A. recommendation by doctors and nurses

B. recommendation by relatives and friends

C. good reputation

D. free

E. not applicable

F. simple and convenient to use

G. others

1. What help would you like to get from the health apps?

A. One week's blood pressure trend

B. one week medication

C. self-blood pressure control evaluation

D. hypertension knowledge

E. patients’ communication

F. communication with doctor if necessary
